# Effectiveness of Virtual Reality-Based Rehabilitation on Balance, Gait, and Functional Independence in Patients With Parkinson’s Disease: Implications for Healthy Longevity

**DOI:** 10.7759/cureus.116247

**Published:** 2026-09-14

**Authors:** Shruti Mahato, Ramendra K Raman, Dhiren K Panda

**Affiliations:** 1 Physiotherapy, School of Health &amp; Allied Sciences, Sanskaram University, Jhajjar, IND; 2 Clinical Anatomy, Sanskaram School of Medical Sciences, Sanskaram University, Jhajjar, IND; 3 Anatomy, Sudha Medical College &amp; Hospital, Kota, IND

**Keywords:** balance, fall risk, functional independence, gait speed, healthy ageing, longevity medicine, parkinson’s disease, rehabilitation, virtual reality

## Abstract

Background and objective: Parkinson’s disease is a progressive neurodegenerative disorder associated with balance impairment, gait dysfunction, falls, disability, and progressive loss of functional independence. Healthy longevity emphasises the preservation of functional ability and well-being rather than survival alone. Virtual reality-based rehabilitation provides an interactive, technology-assisted approach to balance and gait training, but comparative evidence addressing multiple functional domains simultaneously remains limited. This study aims to compare the effectiveness of virtual reality-based rehabilitation with conventional physiotherapy on balance, functional mobility, gait parameters, fall-risk reclassification, adherence, and satisfaction in adults with idiopathic Parkinson’s disease, and to interpret these findings within a healthy-longevity and functional-independence framework.

Methods: This retrospective, multiclinic comparative cohort study reviewed clinical records of 250 adults aged 50 to 60 years with idiopathic Parkinson’s disease and Hoehn and Yahr stage II-III. Participants comprised 125 individuals who received an eight-week virtual reality-based rehabilitation programme and 125 who received conventional physiotherapy. Balance was assessed using the Berg Balance Scale (BBS), functional mobility using the Timed Up and Go (TUG) test, and gait performance using gait speed, stride length, and cadence. Fall-risk categories, treatment adherence, and participant satisfaction were also evaluated. Within-group changes were assessed using paired-samples t-tests, while between-group differences in change scores were assessed using independent-samples t-tests with Cohen’s d as the effect-size measure. Categorical fall-risk changes were assessed using chi-square tests.

Results: Groups were comparable at baseline for age, sex, Hoehn and Yahr stage, disease duration, comorbidity, BBS, TUG, gait speed, and stride length. Baseline cadence differed significantly between groups. Virtual reality-based rehabilitation produced significantly greater improvements than conventional physiotherapy in BBS score (+8.97 ± 1.27 vs +4.52 ± 1.10 points; d=3.75), TUG performance (−3.51 ± 0.54 vs −1.79 ± 0.47 seconds; d=−3.42), gait speed (+0.234 ± 0.031 vs +0.111 ± 0.029 m/s; d=4.10), stride length (+0.141 ± 0.018 vs +0.081 ± 0.020 m; d=3.18), and cadence (+8.17 ± 1.01 vs +4.17 ± 0.89 steps/min; d=4.21; all p<0.001). A lower fall-risk category was achieved by 108 of 125 participants (86.4%) in the virtual reality group compared with 34 of 125 (27.2%) in the conventional physiotherapy group (χ²=86.87, p<0.001). Adherence and satisfaction were also higher in the virtual reality group (94.7 ± 3.1% vs 88.3 ± 3.0%, d=2.14; and 9.03 ± 0.64 vs 7.15 ± 0.53, d=3.18; both p<0.001).

Conclusions: Virtual reality-based rehabilitation was associated with substantially greater short-term improvements in balance, functional mobility, gait, and fall-risk classification than conventional physiotherapy. The findings support virtual reality-based rehabilitation as a potentially scalable strategy for preserving mobility and functional independence in people with Parkinson’s disease. Because the study did not evaluate survival or long-term disability, the healthy-longevity implications should be regarded as a healthspan-orientated hypothesis requiring prospective longitudinal evaluation.

## Introduction

Parkinson’s disease is a progressive neurodegenerative disorder with a rapidly increasing global burden. The Global Burden of Disease Study 2021 estimated that 11.77 million people worldwide were living with Parkinson’s disease in 2021, representing a substantial increase over the preceding decades [1]. A recent modelling study projected that the global number of people living with Parkinson’s disease may reach approximately 25.2 million by 2050, largely driven by population ageing [2]. This growing burden has important implications for strategies designed not only to manage disease symptoms but also to preserve functional capacity over the course of ageing.

Balance impairment and gait dysfunction are among the most clinically consequential manifestations of Parkinson’s disease. Postural instability, reduced gait speed, shortened stride length, impaired turning, and altered cadence can restrict mobility and increase vulnerability to falls. Falls are common during the course of Parkinson’s disease and can contribute to injury, fear of falling, reduced physical activity, deconditioning, and progressive dependence [3]. Consequently, rehabilitation strategies that preserve balance and mobility have an important role in maintaining independence.

The World Health Organization defines healthy ageing as the process of developing and maintaining functional ability that enables well-being in older age [4]. Functional ability includes mobility, the ability to meet basic needs, participation in relationships and society, and the capacity to make decisions and continue meaningful activities. Within this framework, healthy longevity is not synonymous with lifespan alone. Preservation of mobility and independence represents an important component of the quality of additional years of life. Gait speed has particular relevance to this concept. A pooled analysis of 34,485 community-dwelling older adults demonstrated that gait speed was independently associated with survival, with each 0.1 m/s increase associated with a lower mortality hazard [5]. This does not establish that increasing gait speed through rehabilitation extends life, but it supports gait speed as a clinically meaningful marker of overall functional and health status.

Virtual reality-based rehabilitation has emerged as a technology-assisted approach to neurological rehabilitation. Virtual reality systems can provide task-specific practice, real-time visual or auditory feedback, progressively challenging environments, and goal-directed activities. These characteristics may enhance motor learning and engagement while allowing balance and gait exercises to be delivered in an interactive format.

Previous clinical trials have reported beneficial effects of virtual reality-based interventions on postural instability, activities of daily living (ADL), walking ability, balance, and other motor outcomes in people with Parkinson’s disease [6-9]. The V-TIME (Virtual Reality-Treadmill Training Program Augmented by Virtual Reality to Decrease Fall Risk in Older Adults) randomised trial also demonstrated that adding a non-immersive virtual reality component to treadmill training could reduce fall risk in older adults, with particularly relevant effects among participants with Parkinson’s disease [10].

A systematic review and meta-analysis involving 1,031 participants reported beneficial effects of virtual reality rehabilitation on balance and gait-related outcomes in Parkinson’s disease [11]. More recently, a dose-response meta-analysis of 32 randomised trials involving 547 participants reported a pooled Berg Balance Scale (BBS) improvement of 3.63 points and identified potentially useful parameters for virtual reality training dose [12]. These findings support the clinical relevance of virtual reality rehabilitation while also demonstrating heterogeneity in platforms, intervention duration, treatment intensity, and outcome selection.

Most previous studies have focused on individual functional domains, whereas real-world independence depends on the interaction of balance, gait, mobility, and fall resistance. Furthermore, relatively few studies have explicitly interpreted rehabilitation outcomes through a healthy-longevity framework. The present study therefore examined multiple functional outcomes simultaneously in a comparatively large real-world sample of 250 participants.

The objective of this study was to compare virtual reality-based rehabilitation with conventional physiotherapy on balance (primary outcome: BBS [13]), functional mobility (primary outcome: Timed Up and Go (TUG) test [14]), gait parameters and categorical fall-risk classification (secondary outcomes), and adherence and satisfaction (exploratory outcomes) among adults with Hoehn and Yahr stage II-III idiopathic Parkinson’s disease [15]. Findings were interpreted within a functional-ability and healthy-longevity framework, recognising that survival, disability-free survival, and long-term functional independence were not directly measured.

## Materials and methods

Study design and setting

This is a retrospective, multi-clinic, comparative cohort study coordinated through the Department of Physiotherapy, School of Health & Allied Sciences, Sanskaram University (Jhajjar, HR, IND). Data were compiled from clinical records of adults with idiopathic Parkinson’s disease who had received either virtual reality-based rehabilitation or conventional physiotherapy as part of routine care at participating physiotherapy clinics. Structured baseline and post-intervention assessment visits were conducted as part of routine clinical care from January to August 2026; the present study subsequently analysed those records. Because this is a retrospective analysis of routinely collected clinical data rather than a prospectively registered controlled trial, formal clinical trial registration was not applicable. The study was approved by the Institutional Ethics Committee of Sanskaram University (approval no. SU/IEC/2026/021). Written informed consent for the clinical use and research use of assessment data was obtained from all participants in accordance with the Declaration of Helsinki.

Participants

Adults aged 50 to 60 years with a clinical diagnosis of idiopathic Parkinson’s disease, Hoehn and Yahr stage II or III, and the ability to ambulate independently for at least 10 metres were eligible for inclusion. Exclusion criteria included atypical or secondary parkinsonism, severe cognitive impairment precluding task comprehension, uncorrected visual or vestibular disorders, and orthopaedic or cardiovascular conditions contraindicating standing exercise. Records of 250 eligible patients were identified: 125 who had received virtual reality-based rehabilitation and 125 who had received conventional physiotherapy. Choice of intervention was determined by the treating clinician and clinic-level programme availability rather than by randomisation; this non-randomised allocation is addressed directly in the limitations.

Interventions

Both groups received supervised, individually delivered sessions over an eight-week intervention period as part of routine clinical care at participating clinics (typically three 45- to 60-minute sessions per week). The virtual reality group trained on a non-immersive, camera-based virtual reality platform incorporating balance-reaching, obstacle-negotiation, and stepping tasks with real-time visual and auditory feedback and progressive task difficulty calibrated to individual performance. The conventional physiotherapy group received a standard, evidence-based programme comprising balance re-education (static and dynamic balance exercises), gait training (cueing and dual-task walking), strengthening (lower-limb progressive resistance), and flexibility exercises, broadly matched to the virtual reality group for session frequency, duration, and progressive difficulty. Outcome measures were recorded by the treating clinical team as part of standard assessment; formal blinding of assessors was not part of this retrospective, real-world data collection and is noted as a limitation.

Outcome measures

Balance was assessed with the BBS (0-56 points; higher points indicate better balance), a validated and widely used clinical balance measure in Parkinson’s disease [13]. Functional mobility and fall risk were assessed with the TUG test (measured in seconds; fewer seconds indicate better mobility), a validated predictor of falling in Parkinson’s disease [14]. Fall-risk categories were defined using standard TUG cut-offs commonly applied in clinical practice for older adults and Parkinson’s disease populations: high risk (>13.5 seconds), moderate risk (11.0-13.5 seconds), and low risk (<11.0 seconds), supplemented by clinical judgement. Gait parameters include gait speed (m/s), stride length (m), and cadence (steps/min) and were captured using an instrumented gait analysis system (GAITRite electronic walkway (CIR Systems Inc., Franklin, NJ, USA) or equivalent clinic-standard system). Participants completed three walking trials at comfortable speed over a 6 m to 8 m instrumented pathway; the mean of the three trials was used for analysis. Treatment adherence (percentage of scheduled sessions completed, including documented make-up sessions) and participant satisfaction (0-10 numerical rating scale) were recorded at the end of the intervention period. All outcomes were assessed at baseline and immediately post-intervention by assessors following a standardised protocol.

Statistical analysis

Data were analysed in Python (SciPy, pandas; Python Software Foundation, Wilmington, DE, USA). Baseline comparability between groups was assessed using independent-samples t-tests for continuous variables and chi-square tests for categorical variables. Normality of continuous variables was assessed using the Shapiro-Wilk test and visual inspection of histograms/Q-Q plots. For each continuous outcome, within-group pre-post change was assessed using paired t-tests, and between-group differences in change scores were assessed using independent-samples t-tests. Cohen’s d was calculated using the pooled standard deviation of the change scores. A confidence interval of 95% was calculated for between-group mean differences and effect sizes. Because baseline cadence differed significantly between groups, an analysis of covariance (ANCOVA) was performed with post-intervention cadence as the dependent variable and baseline cadence as the covariate; sensitivity analyses using change scores adjusted for baseline were also conducted for other gait parameters. Fall-risk category shift (improved vs not improved) and post-intervention category distribution were compared between groups using chi-square tests; McNemar’s test was used for paired categorical transitions within groups. A transition matrix of individual-level fall-risk category changes was constructed. Pearson correlation examined the relationship between treatment adherence and outcome change within the virtual reality group. A pre-specified subgroup analysis compared the treatment effect on BBS change between Hoehn and Yahr stage II and stage III participants, including a group × stage interaction term. Two-sided p < 0.05 was considered statistically significant throughout. No formal a priori sample-size calculation was performed because the study was retrospective; the available sample of 250 participants provided high precision for the observed effect sizes.

## Results

Participant characteristics and baseline comparability

A total of 250 participants completed baseline and post-intervention assessments, including 125 participants in the virtual reality group and 125 in the conventional physiotherapy group. The groups were generally comparable at baseline across demographic and clinical characteristics (age, sex, Hoehn and Yahr stage, disease duration, comorbidity, BBS, TUG test, gait speed, and stride length). A statistically significant between-group difference was observed in baseline cadence (101.37 ± 3.17 steps/min in the virtual reality group vs 102.35 ± 2.86 steps/min in the conventional physiotherapy group; p = 0.011) (Table 1).

**Table 1:** Baseline characteristics of participants by treatment group *Statistically significant at p<0.05; BBS: Berg Balance Scale, TUG: Timed Up and Go

Characteristic	Virtual reality (n=125)	Conventional physiotherapy (n=125)	p-value
Age (years)	54.6 ± 3.1	55.0 ± 3.0	0.246
Sex (female/male)	68/57	56/69	0.164
Hoehn and Yahr stage II/III	57/68	65/60	0.376
Disease duration (years)	6.65 ± 1.90	6.77 ± 1.90	0.604
Comorbidity present (n (%))	57 (45.6)	62 (49.6)	0.613
Baseline BBS points	41.89 ± 3.06	41.75 ± 2.88	0.721
Baseline TUG test (seconds)	14.78 ± 1.05	14.77 ± 1.02	0.932
Baseline gait speed (m/s)	0.894 ± 0.057	0.900 ± 0.059	0.403
Baseline stride length (m)	1.002 ± 0.054	1.002 ± 0.051	0.981
Baseline cadence (steps/min)	101.37 ± 3.17	102.35 ± 2.86	0.011*

Balance and functional mobility

Both treatment groups demonstrated significant within-group improvement from baseline in BBS and TUG test performance (within-group p < 0.001 for both outcomes). The magnitude of improvement was substantially greater in the virtual reality group. The BBS score increased by 8.97 ± 1.27 points in the virtual reality group compared with 4.52 ± 1.10 points in the conventional physiotherapy group (between-group difference 4.45 points, 95% CI 4.15-4.75; p < 0.001; Cohen’s d = 3.75). The TUG test performance decreased by 3.51 ± 0.54 seconds in the virtual reality group compared with a reduction of 1.79 ± 0.47 seconds in the conventional physiotherapy group (between-group difference −1.72 seconds, 95% CI −1.85 to −1.59; p < 0.001; Cohen’s d = −3.42) (Table 2).

**Table 2:** Pre-post changes in balance, functional mobility, and gait outcomes BBS: Berg Balance Scale, TUG: Timed Up and Go

Outcome	Virtual reality change, mean ± SD	Conventional physiotherapy change, mean ± SD	Between-group p-value	Cohen's d
BBS points	+8.97 ± 1.27	+4.52 ± 1.10	<0.001	3.75
TUG test (seconds)	−3.51 ± 0.54	−1.79 ± 0.47	<0.001	−3.42
Gait speed (m/s)	+0.234 ± 0.031	+0.111 ± 0.029	<0.001	4.10
Stride length (m)	+0.141 ± 0.018	+0.081 ± 0.020	<0.001	3.18
Cadence (steps/min)	+8.17 ± 1.01	+4.17 ± 0.89	<0.001	4.21

Gait parameters

Gait speed, stride length, and cadence improved significantly within both treatment groups (all within-group p < 0.001). The virtual reality group demonstrated significantly greater improvement than the conventional physiotherapy group for each gait parameter. After baseline adjustment using ANCOVA, the between-group difference in post-intervention cadence remained statistically significant and of similar magnitude. Results for gait speed and stride length were essentially unchanged in sensitivity analyses adjusting for baseline values (Figure 1).

**Figure 1:**
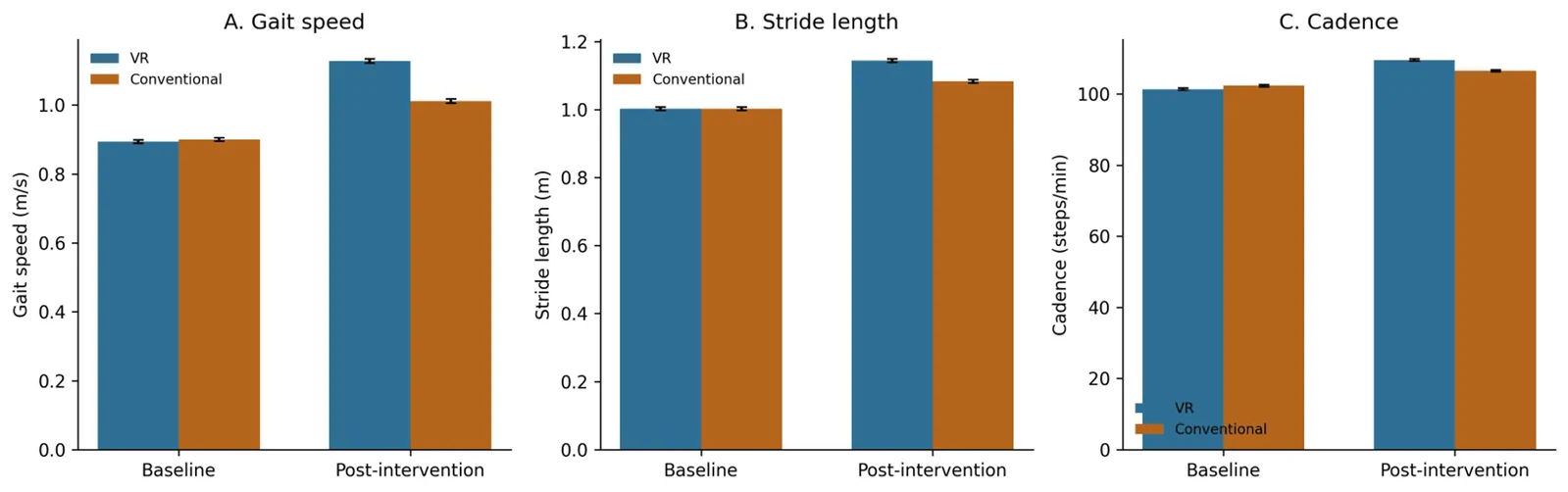
Changes in gait parameters following the eight-week intervention Gait speed (A), stride length (B), and cadence (C) at baseline and post-intervention by group. Error bars represent the standard error of the mean.

Fall-risk reclassification

At baseline, the two groups had identical fall-risk distributions, with 66 participants classified as high risk, 59 as moderate risk, and none as low risk in each group. Following the intervention, 108 of 125 participants in the virtual reality group (86.4%) shifted to a lower fall-risk category, and no participant in the virtual reality group remained in the high-risk category at the time of post-intervention assessment. The post-intervention distribution in the virtual reality group was 0 = high risk, 42 = moderate risk, and 83 = low risk. In the conventional physiotherapy group, 34 participants (27.2%) improved their risk category, whereas 34 participants (27.2%) shifted to a higher-risk category; the post-intervention distribution remained 66 = high-risk, 59 = moderate-risk, and 0 = low-risk. The between-group difference in the proportion of participants improving their fall-risk category was statistically significant (χ² = 86.87, p < 0.001). The difference in post-intervention category distribution was also statistically significant (χ² = 151.86, p < 0.001). An individual-level transition matrix is provided in Table 3. McNemar’s test confirmed significant within-group improvement in the virtual reality group (p < 0.001) but not in the conventional group.

**Table 3:** Fall-risk category distribution and reclassification by treatment group

Group	Baseline high/moderate/low	Post-intervention high/moderate/low	Improved category, n (%)
Virtual reality (n=125)	66/59/0	0/42/83	108 (86.4%)
Conventional physiotherapy (n=125)	66/59/0	66/59/0	34 (27.2%)

Adherence, satisfaction, and subgroup consistency

Treatment adherence was significantly higher in the virtual reality group than in the conventional physiotherapy group (94.7 ± 3.1% vs 88.3 ± 3.0%; p < 0.001; Cohen’s d = 2.14). Participant satisfaction was also significantly higher in the virtual reality group (9.03 ± 0.64 vs 7.15 ± 0.53; p < 0.001; Cohen’s d = 3.18). Within the virtual reality group, adherence demonstrated a weak inverse correlation with the TUG test improvement (r = −0.178, p = 0.047); the magnitude of this association was small and should be interpreted cautiously. No significant correlation was observed between adherence and changes in the BBS score, gait speed, stride length, or cadence (all p > 0.09).

The magnitude of the BBS benefit was consistent across the Hoehn and Yahr stage II and stage III subgroups. Participants with stage II disease demonstrated an improvement of +9.07 points in the virtual reality group compared with +4.54 points in the conventional group (p < 0.001). Among participants with stage III disease, improvement was +8.89 points in the virtual reality group compared with +4.49 points in the conventional group (p < 0.001). The group × stage interaction term was not statistically significant (p = 0.72), supporting consistency of the treatment effect across disease severity.

## Discussion

This retrospective multiclinic comparative study of 250 adults with Hoehn and Yahr stage II-III Parkinson’s disease [15] found that virtual reality-based rehabilitation was associated with substantially greater short-term improvements than conventional physiotherapy across balance, functional mobility, gait, and fall-risk outcomes, with higher adherence and satisfaction and consistent benefit across disease-severity subgroups. These findings are considered below in relation to the existing evidence base, their implications for healthy longevity, and the limitations that should guide their interpretation.

Comparison with existing evidence

These findings align directionally with the existing evidence base: previous trials have reported benefit for postural instability, ADL, walking ability, and balance [6-9], and adding virtual reality to treadmill training has reduced fall risk in older adults, with particular benefit in Parkinson’s disease [10]. A meta-analysis of 1,031 participants supported benefit for balance and gait outcomes [11], and a more recent dose-response meta-analysis reported a pooled BBS improvement of 3.63 points [12]. The magnitude of improvement observed in the present cohort appeared larger than that reported in some previous syntheses; however, direct comparison is difficult because studies differed in design, comparator, intervention dose, outcome definitions, and effect-estimation methods. The unusually large effect sizes observed here (Cohen’s d approximately 3.2-4.2) warrant cautious interpretation and may partly reflect selection factors, measurement characteristics, or unmeasured confounding inherent in a non-randomized design.

Implications for healthy longevity

The World Health Organization frames functional ability, not disease absence, as the central marker of healthy ageing [4], and gait speed is an independently validated predictor of survival in older adults [5]. The virtual reality group’s gains in gait speed, balance, and fall-risk reclassification are therefore healthspan-relevant functional surrogates rather than direct evidence of extended survival or reduced long-term disability. The findings should be understood as evidence for a healthspan-orientated rehabilitation strategy rather than a demonstrated life-extending intervention.

Strengths and limitations

Strengths include the comparatively large real-world multiclinic sample of 250 participants and the simultaneous assessment of balance, gait, and fall-risk domains within an explicit healthy-longevity framework. The principal limitation is the retrospective, non-randomised design: treatment was determined by clinician and clinic-level availability rather than randomisation, so confounding by indication cannot be excluded, and the observed differences cannot establish that virtual reality itself caused the superior outcomes. Although most baseline characteristics were well balanced, cadence differed significantly between groups at baseline; therefore, the cadence findings should be interpreted with particular caution even after baseline-adjusted analyses. Additional limitations include heterogeneity introduced by multi-clinic data collection, a narrow age and disease-severity range limiting generalisability, assessment at only one post-intervention time point, and adherence and satisfaction data drawn from clinical records. Higher adherence and satisfaction in the virtual reality group may have contributed to the observed functional differences and therefore represent potential mediators or confounders. A small number of adherence values exceeded 100%, reflecting completion of make-up sessions, and are reported as documented according to the predefined definition (completed sessions including make-ups divided by scheduled sessions). Scalability was not directly evaluated; implementation cost, equipment availability, therapist training, technical requirements, home deployment, patient access, clinic infrastructure, and cost-effectiveness were not assessed.

Future directions

Prospective, randomised, multicentre trials with standardised protocols, blinded assessment, and longitudinal follow-up, including objectively recorded falls, disability, and functional-independence trajectories, are needed to confirm these findings and directly test their relevance to healthy longevity. Such studies should also evaluate whether the observed gains are sustained after supervised treatment ends and whether virtual reality rehabilitation can be feasibly implemented in routine and home-based care.

## Conclusions

In this retrospective multiclinic comparative cohort of 250 adults with Parkinson’s disease, virtual reality-based rehabilitation was associated with significantly greater short-term improvements than conventional physiotherapy in balance, functional mobility, gait speed, stride length, cadence, and fall-risk classification. Higher adherence and satisfaction were also observed in the virtual reality group, and the balance benefit was consistent across Hoehn and Yahr stage II and stage III disease. These findings support virtual reality-based rehabilitation as a potentially useful strategy for preserving mobility and functional independence in people living with Parkinson’s disease. The healthy-longevity relevance of the findings should be understood in terms of healthspan and preservation of functional ability rather than demonstrated extension of lifespan. Prospective randomized studies with long-term follow-up are required to determine whether improvements in balance, gait, and fall-risk status translate into sustained independence, reduced disability, fewer falls, and ultimately improved long-term health outcomes.
